# ICAM1 (CD54) Contributes to the Metastatic Capacity of Gastric Cancer Stem Cells

**DOI:** 10.3390/ijms25168865

**Published:** 2024-08-14

**Authors:** José Manuel Tinajero-Rodríguez, Lizbeth Ramírez-Vidal, Jared Becerril-Rico, Eduardo Alvarado-Ortiz, Dámaris P. Romero-Rodríguez, Fernando López-Casillas, Daniel Hernández-Sotelo, Fernando Fernández-Ramírez, Adriana Contreras-Paredes, Elizabeth Ortiz-Sánchez

**Affiliations:** 1 Subdirección de Investigación Básica, Instituto Nacional de Cancerología, Av. San Fernando 22, Colonia Sección XVI, Mexico City 14080, Mexico; jtinajeror@outlook.es (J.M.T.-R.); adrycont@yahoo.com.mx (A.C.-P.); 2 Facultad de Ciencias Químico Biológicas, Universidad Autónoma de Guerrero, Chilpancingo 39090, Mexico; danhs1mx@yahoo.com; 3 Posgrado de Ciencias Biomédicas, Facultad de Medicina, Universidad Nacional Autónoma de México, Circuito Exterior s/n, Ciudad Universitaria, Mexico City 04510, Mexico; lisbet_rv@hotmail.com; 4 Posgrado en Ciencias Biológicas, Universidad Nacional Autónoma de México, Mexico City 04510, Mexico; jared.becerril.rico@gmail.com (J.B.-R.); eduralv.o@gmail.com (E.A.-O.); 5 Laboratorio Nacional Conahcyt de Investigación y Diagnóstico por Inmunocitofluorometría (LANCIDI), INER, Mexico City 14080, Mexico; unidad.citometria.iner@gmail.com; 6 Instituto de Fisiología Celular, Universidad Nacional Autónoma de México, Circuito Exterior s/n, Ciudad Universitaria, Mexico City 04510, Mexico; fcasilla@ifc.unam.mx; 7 Unidad de Genética, Hospital General de México, Mexico City 06720, Mexico; ffernandez@ciencias.unam.mx

**Keywords:** gastric cancer, cancer stem cell, metastasize, tumorigenic

## Abstract

Gastric cancer is the fourth leading cause of cancer deaths worldwide. The presence of chemoresistant cells has been used to explain this high mortality rate. These higher tumorigenic and chemoresistant cells involve cancer stem cells (CSCs), which have the potential for self-renewal, a cell differentiation capacity, and a greater tumorigenic capacity. Our research group identified gastric cancer stem cells (GCSCs) with the CD24+CD44+CD326+ICAM1+ immunophenotype isolated from gastric cancer patients. Interestingly, this GCSC immunophenotype was absent in cells isolated from healthy people, who presented a cell population with a CD24+CD44+CD326+ immunophenotype, lacking ICAM1. We aimed to explore the role of ICAM1 in these GCSCs; for this purpose, we isolated GCSCs from the AGS cell line and generated a GCSC line knockout for ICAM1 using CRISPR/iCas9, which we named GCSC-ICAM1^KO^. To assess the role of ICAM1 in the GCSCs, we analyzed the migration, invasion, and chemoresistance capabilities of the GCSCs using in vitro assays and evaluated the migratory, invasive, and tumorigenic properties in a zebrafish model. The in vitro analysis showed that ICAM1 regulated STAT3 activation (pSTAT3-ser727) in the GCSCs, which could contribute to the ability of GCSCs to migrate, invade, and metastasize. Interestingly, we demonstrated that the GCSC-ICAM1^KO^ cells lost their capacity to migrate, invade, and metastasize, but they exhibited an increased resistance to a cisplatin treatment compared to their parental GCSCs; the GCSC-ICAM1^KO^ cells also exhibited an increased tumorigenic capability in vivo.

## 1. Introduction

Gastric cancer (GC) is one of the most frequently detected cancers worldwide [1]; considering public health, GC ranks fifth in incidence and fourth in mortality around the world [2,3]. One explanation for GC’s high mortality rate is the presence of a low subpopulation of radio- and chemoresistant cells called cancer stem cells (CSCs) [4]. CSCs have been proposed as a therapeutic target, but the principal challenge is their identification and isolation from tumors, due to their scarcity. Recently, the identification and isolation of cancer stem cells from solid tumors has been the subject of several research studies [5,6,7,8], as cancer stem cells have been proposed as therapeutic targets for the treatment of different types of cancer [9,10,11]. Identifying these cells is possible because of their surface markers, which can be shared with their non-tumorigenic counterparts [12,13]. In our group, we identified a gastric cancer stem cell (GCSC) subpopulation with the CD24+CD44+CD326+ICAM1+ immunophenotype in patients with gastric cancer. In contrast, the evaluation of GCSCs from non-tumor tissue samples showed that the cell surface marker ICAM1 was absent [14].

Intercellular adhesion molecule 1 (ICAM-1 or CD54) is a surface glycoprotein with five immunoglobulin domains that are essential for the transendothelial migration of lymphocytes, a transmembrane domain, and threonine-rich cytoplasmic domains [15,16]. ICAM1 is expressed at low levels in various cell types, including leukocytes, epithelial and endothelial cells, fibroblasts, and keratinocytes [17,18]. Meanwhile, their expression increases substantially in the presence of inflammatory regulators such as INF-γ, IL-1β, TNF-α [19], bacterial lipopolysaccharides [20], and phorbol esters (PMAs) [21]. In addition, the upregulation of ICAM1 has been demonstrated in different types of cancer, including lung adenocarcinoma, gastric cancer, breast cancer, melanoma, and oral squamous cell carcinoma, among others [16]. ICAM-1 expression has been correlated with the aggressiveness and metastasis of non-small-cell lung carcinoma [22]. The oncogenic role of ICAM1 in colorectal cancer was recently reported using in vivo and in vitro analyses to demonstrate the migratory, invasive, and angiogenic potential of ICAM1-expressing cells [23]. Because of the relationship between ICAM1 and its ability to promote EMT mechanisms, as well as the stemness, through its ability to activate the transcription factor STAT3 [23], ICAM1 has been identified as a potential therapeutic target for cancer stem cells (CSCs), and it has been proposed as a target in immunotherapy. While this project shed light on the significance of CSCs in driving cancer progression, it also highlighted the challenges associated with using CSCs as viable therapeutic targets. These complexities require further research and understanding to develop effective strategies for targeting CSCs to improve cancer treatment efficacy.

## 2. Results

### 2.1. CRISPR/iCas9 Genome Editing of ICAM1 in Gastric Cancer Stem Cells

**Figure 1:**
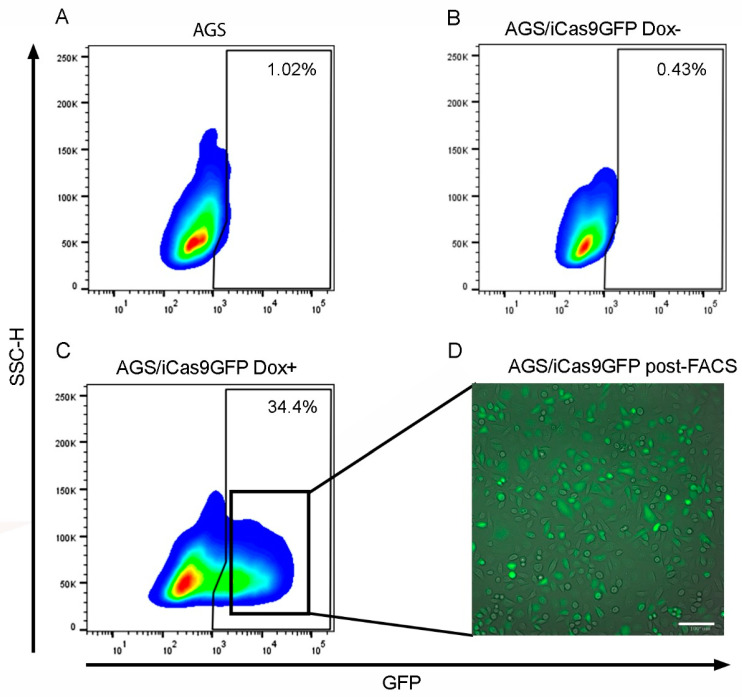
The generation of an AGS/iCas9GFP cell line. (**A**) GFP basal expression in the AGS cell line. (**B**) GFP expression in the AGS/iCas9GFP cell line Dox−. (**C**) GFP expression in the AGS/iCas9GFP cell line after inducing with Dox+. (**D**) The fluorescence microscopy of the AGS/iCas9GFP cell lines after FACS, 20x. Dox+ and Dox−, with or without doxycycline, respectively. Bars indicate 100 µm.

Previously, our research group reported a GCSC immunophenotype (CD24+CD44+CD326+ICAM1+) in gastric cancer patients. We demonstrated the ability of these cells to migrate and metastasize in zebrafish larvae [14]. Interestingly, these cells were not present in healthy patients, but we were able to find a cell subpopulation with the CD24+CD44+CD326+ICAM1− immunophenotype [14]. To assess the biological function of ICAM1 in GCSCs, we used the CRISPR/iCas9 system to generate an ICAM1 knockout gastric cancer stem cell line (GCSC-ICAM1^KO^). The AGS cell line was transfected with the plasmid Lenti-iCas9-neo, which has a selectable cassette harboring an inducible green fluorescent protein (GFP) and a selection drug (neomycin). The basal GFP expression in the AGS cell line was 1.02% (Figure 1A). We also determined the basal GFP expression in the AGS cell line transfected with the plasmid Lenti-iCas9-neo but without doxycycline (Figure 1B), which was 0.43%. We obtained 34.4% of the GFP+ cells by adding doxycycline to the Lenti-iCas9-neo-transfected cells (Figure 1C). To obtain the AGS/iCas9GFP cell line, we isolated GFP-positive cells (black squares, Figure 1C) using FACS and by maintaining the cells under adherent conditions in a 10% FBS F12 medium supplemented with neomycin. To verify the phenotype of the isolated cells, we induced GFP expression by adding doxycycline to the culture and observed the cells using fluorescence microscopy (Figure 1D).

**Figure 2:**
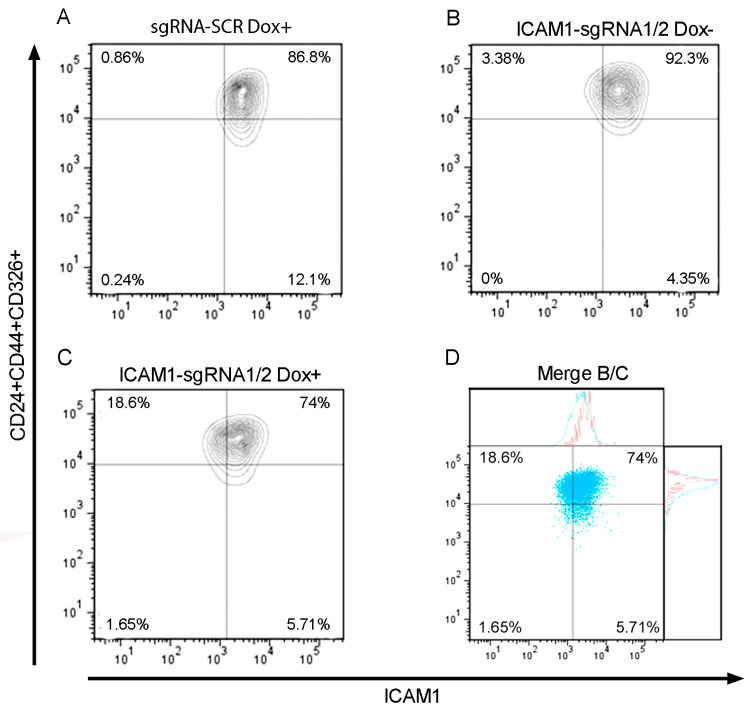
GCSC/ICAM1^KO^ transduction efficiency 48 h post-induction. (**A**) AGS/iCas9GFP transduced with sgRNA-SCR Dox+. (**B**) AGS/iCas9GFP transduced with ICAM1-sgRNA 1/2 Dox−. (**C**) AGS/iCas9GFP transduced with ICAM1-sgRNA 1/2 Dox+. (**D**) Merge between blot B (red population) and blot C (blue population) panels. Dox (doxycycline).

The cell line AGS/iCas9GFP was co-transfected with the pICAM1/1CROPseq and pICAM1/2CROPseq plasmids. We generated a control cell group transfected with pSCR-CROPseq, which showed no change in the percentage of ICAM1-negative cells (ICAM1^−^) after the induction with doxycycline (Figure 2A). We analyzed the cells co-transfected with the plasmids pICAM1/1CROPseq and pICAM1/2CROPseq without doxycycline induction, and we observed 3.3% CD24+CD44+CD326+ICAM1− cells (Figure 2B). When this cell line was induced with doxycycline for 48 h, we obtained GCSC-ICAM1^KO^ cells with approximately a 15% efficiency (Figure 2C). In Figure 2D, we show the merge between items B and C. The cells were obtained through FACS and were maintained in a knockout medium supplemented with neomycin and puromycin (see Section 4).

**Figure 3:**
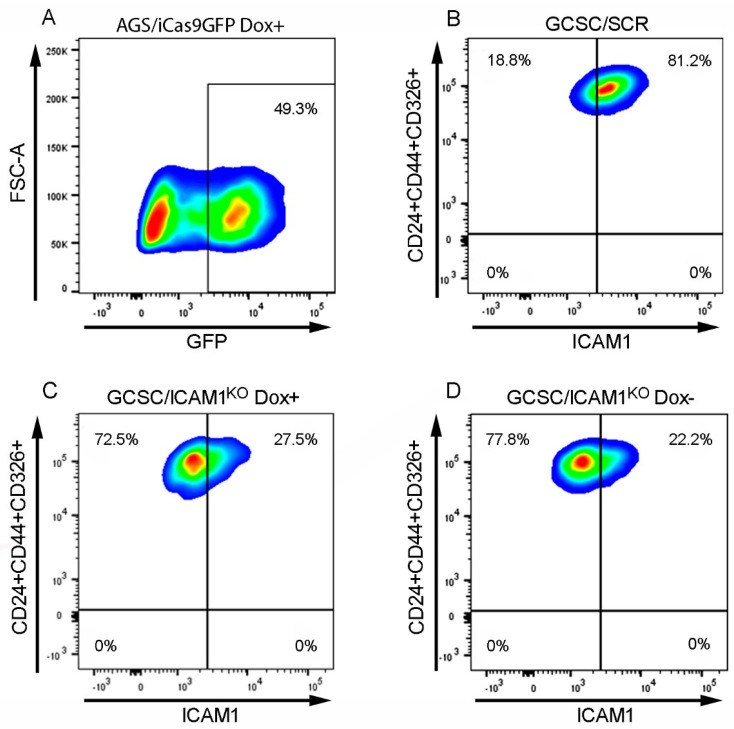
The CRISPRiCas9 generation of a stable GCSC/ICAM1^KO^ cell line. (**A**) GFP expression in AGS/iCas9GFP Dox+ cells. (**B**) GCSC/SCR Dox+. (**C**) GCSC/ICAM1^KO^ Dox+. (**D**) GCSC/ICAM1^KO^ Dox−. The panels represent pass 10 after cell sorting with the respective immunophenotype. Dox (doxycycline).

After ten passages, we analyzed the stability of the system using FACS. We observed that the cells transfected with pSCR-CROPseq had approximately 80% ICAM1+ cells from the cell subpopulation CD24+CD44+CD326+ after 24 h of doxycycline induction (Figure 3B). In contrast, the cells transfected with both pICAM1/1CROPseq and pICAM1/2CROPseq decreased by approximately 70% in this ICAM1+ subpopulation after 24 h of doxycycline induction (Figure 3C). Interestingly, we demonstrated that an induction with doxycycline would not be necessary in subsequent experiments (Figure 3D).

### 2.2. The ICAM1/pSTAT3 Axis Could Regulate the Transcriptional Factors Associated with Stemness

**Figure 4:**
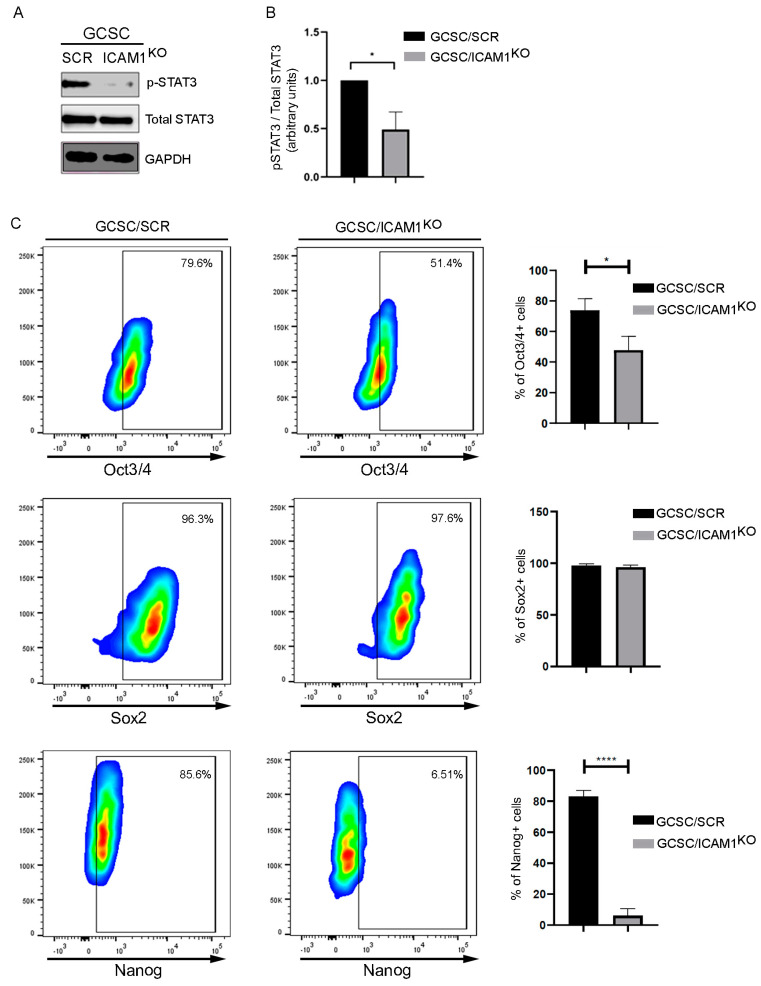
Regulation of stemness-related transcription factor expression by ICAM1/STAT3 axis. (**A**) Western blot analysis of total lysates of GCSC/ICAM1^KO^ and GCSC/SCR cells demonstrated diminished levels of phosphor-STAT3 (p-STAT3-ser727) in GCSC/ICAM1^KO^ cells. (**B**) Densitometry of Western blot * *p* < 0.05. (**C**) Representative dot plot of cytometry flow analysis; GCSC/ICAM1^KO^ cells reduced expression of stem cell markers Oct3/4 and Nanog but not Sox2. * *p* < 0.05. **** *p* < 0.0001.

It has been reported that ICAM1 serves as a scaffold protein for Src phosphorylation and the activation of downstream signaling molecules such as STAT3. Once STAT3 (pSTAT3-ser727) is phosphorylated, it can localize into the nucleus and act as a transcriptional regulator of Nanog, a stemness factor; the localization of pSTAT3 into the nucleus can also generate positive feedback for ICAM1 expression. To determine if ICAM1 can regulate the activation of STAT3 in GCSCs, we analyzed the activation of STAT3 using a Western blot in GCSC/ICAM1^KO^ cells. Our results showed that the activation of STAT3 decreased in the GCSC/ICAM1^KO^ cells, in contrast to the GCSC/SCR cell line (Figure 4A). This result demonstrates that ICAM1 is involved in STAT3 activation, which could be related to the expression of the transcriptional factor associated with stemness. To support this hypothesis, we analyzed the expression of transcriptional factors such as Oct3/4, Sox2, and Nanog in the GCSC/ICAM1^KO^ cells. The expression of Oct3/4 decreased by 20%, and the expression of Nanog decreased by 81% in the GCSC/ICAM1^KO^ cells compared to the GCSC/SCR cells, but we did not observe any changes in the expression of Sox2 (Figure 4C). These results indicate a relationship between ICAM1 and the stemness of GCSCs that is mediated by STAT3 activation.

### 2.3. GCSC/ICAM1^KO^ Cells Exhibited Decreased Migration and Invasion In Vitro

**Figure 5:**
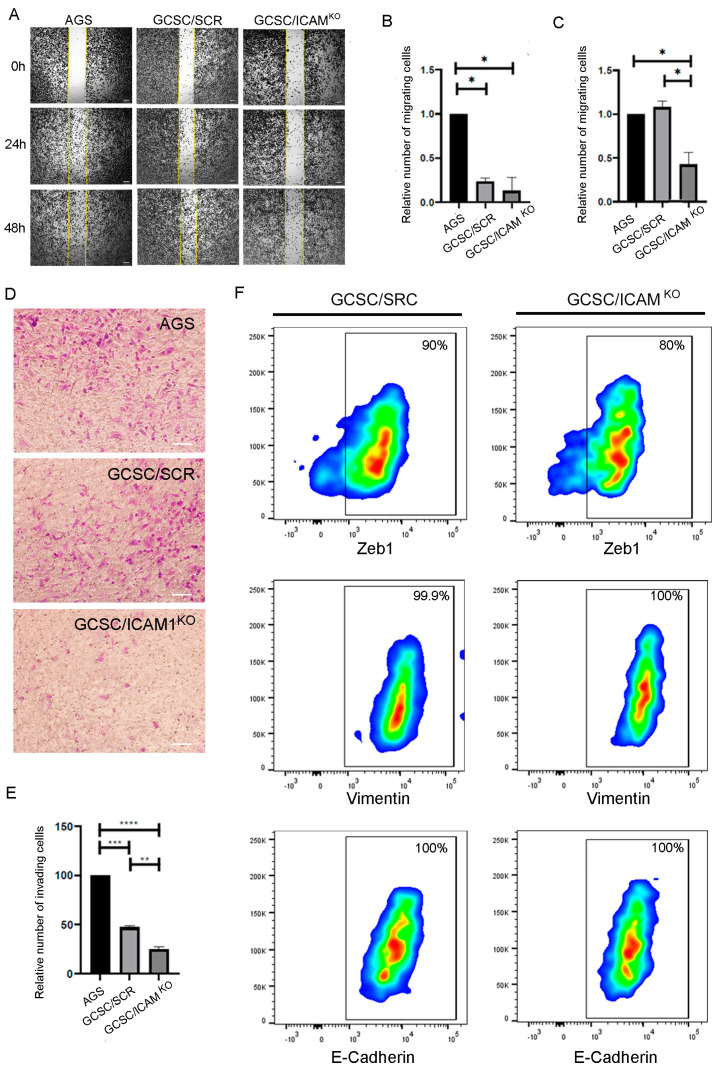
GCSC/ICAM1^KO^ cells lost their ability to migrate and invade but did not alter the expression of EMT markers. (**A**) Representative images of the migration assay; GCSC/ICAM1^KO^ cells exhibited a decreased ability to migrate at 24 h and 48 h, 4X. Bars indicate 200 µm. (**B**,**C**) A quantitative analysis of the relative number of migrating cells represented in (**A**) at 24 (**B**) and 48 h (**C**); n = 3 per group. (**D**) Representative images of the invasion assay; GCSC/ICAM1^KO^ cells exhibited a decreased ability to invade at 24 h, 20X. Bars indicate 100 µm. (**E**) A quantitative analysis of the invasion assay. The values represent the mean ± SD of three independent experiments and are expressed in relative percentages. Asterisks indicate the comparison made between groups. * *p* < 0.05, ** *p* < 0.01, *** *p* < 0.001, and **** *p* < 0.0001. (**F**) A representative dot plot showing the effect of ICAM1 deletion on the expression level of EMT-related proteins.

A relationship between stemness and the EMT has long been proposed; cells with a stem-like phenotype exhibit the ability to migrate and invade. To determine whether GCSC/ICAM1^KO^ affected the ability of the cells to migrate and invade, we analyzed the involvement of ICAM1 in the migration and invasion capacities of GCSCs using wound-healing and transwell assays. Firstly, we determined cell migration using a wound-healing assay. The assay showed that the relative number of migrating GCSC/ICAM1^KO^ cells did not change at 24 h compared to GCSC/SCR cells. However, the relative number of migrating GCSC/ICAM1^KO^ cells decreased compared to the AGS and GCSC/SCR cells at 48 h (Figure 5). These findings indicate that the partial or total elimination of ICAM1 significantly inhibits the migration ability of GCSCs in vitro. Next, we examined the invasion capabilities using a Matrigel assay. The cell invasion assay showed that the GCSC/ICAM1^KO^ cells resulted in a significantly lower proportion of invading cells through the Matrigel-coated chamber than the AGS and GCSC/SCR cells (Figure 5). This result shows that, in vitro, the elimination of ICAM1 decreases the ability of gastric cancer stem cells to migrate and invade.

Our results suggest that ICAM1 expression is a key factor in the acquisition of a mesenchymal-like phenotype. To analyze whether these changes in the migratory and invasive capacity of GCSC/ICAM1^KO^ cells are regulated by the epithelial–mesenchymal transition (EMT), we evaluated the expression of the mesenchymal markers Zeb1 and vimentin and the epithelial marker E-cadherin. The results show no significant changes in their expression (Figure 5). These results suggest that GCSC/ICAM1^KO^ cells do not completely lose their stemness; therefore, under our conditions, ICAM1 did not determine the acquisition of an epithelial or mesenchymal phenotype in GCSCs in vitro.

### 2.4. The Knockout of ICAM1 in GCSCs Decreased Their Migration and Invasion In Vivo

**Figure 6:**
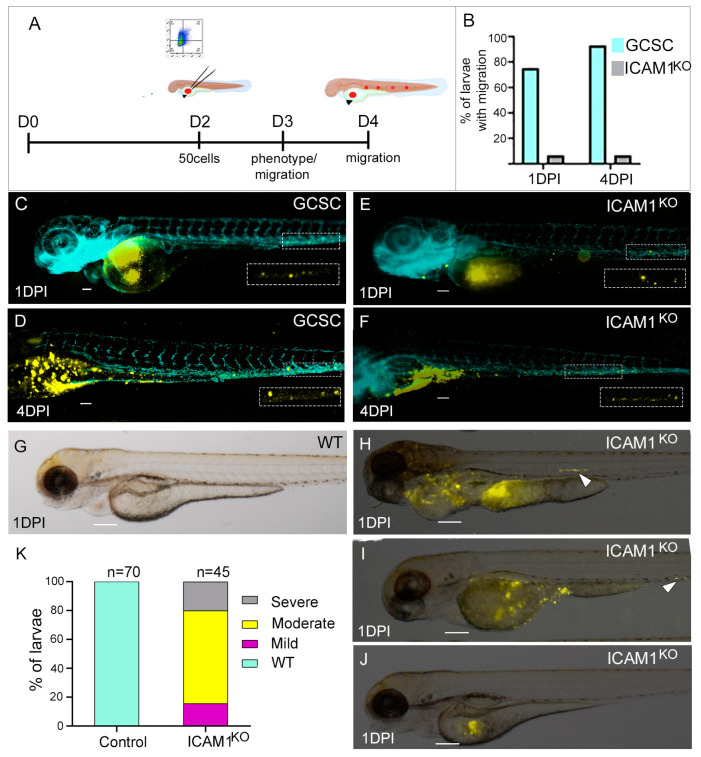
The tumorigenic and migratory capabilities of xenotransplanted GCSC/ICAM1^KO^ cells. (**A**) The experimental design. (**B**) The percentage of embryos with migrating GCSCs or GCSC/ICAM1^KO^ cells after 1 and 4 dpi. (**C**,**D**) show larvae injected with GCSCs after 1 or 4 dpi, respectively. (**E**,**F**) show larvae injected with GCSC/ICAM1^KO^ cells after 1 or 4 dpi, respectively. (**H**–**J**) show larvae after 1 dpi with severe, moderate, or mild phenotypes, respectively. (**G**) shows a wild-type larva. (**K**) shows the percentage of larvae with severe, moderate, or mild phenotypes (n = 45). The images were obtained with a Nikon SMZ1500 stereomicroscope. All the bars indicate 100 µm. GCSC = AGS/GCSC, and ICAM1^KO^ = GCSC/ICAM1^KO^. All boxes and arrow heads indicate sites with migrating cells.

To determine whether ICAM1 ablation in GCSCs affects migration or metastasis in vivo, GCSC-ICAM1^KO^ cells or GCSCs were injected into the yolk of 2 dpf (days post-fertilization) Tg (fli1:EGFP)y1 zebrafish embryos. The migration and metastasis were followed at 1, 4, or 6 dpi (days post-injection) (Figure 6A). After one day of xenotransplantation (n = 60), 75% of the larvae injected with GCSCs showed fluorescently labeled cells in distal portions of the tail, and the percentage increased to 93% at 4 dpi (Figure 6B–E). In contrast, only 6.6% of the larvae xenotransplanted with GCSC/ICAM1^KO^ (CD24+CD44+CD326+ICAM1−) exhibited migrating cells in distal portions of the tail at 1 dpi or 4 dpi (Figure 6B,E,F). These results correlate with the in vitro analyses (Figure 5). 

Interestingly, after 1 dpi, some of the embryos injected with GCSC/ICAM1^KO^ cells exhibited abnormal phenotypes. We classified the phenotypes as severe, moderate, or mild according to the following description. We observed severe phenotypes in 9/45 (20%) embryos, which displayed a large tumor formation at the yolk sac, resulting in the disruption of the zebrafish structures and cardiac edema; 30/45 (66.6%) embryos showed a moderate phenotype, with tumor mass formation in the yolk sac; and 6/45 (13.3%) larvae developed a mild phenotype with a smaller tumor mass formation in the yolk sac. The larvae with a mild-to-moderate phenotype had mild or no yolk sac disruption at 1 dpi (Figure 6H–J). Two of the larvae with a moderate phenotype had migrating GCSC/ICAM1^KO^ cells in their tail (Figure 6H, white arrowhead), and one of the larvae with a severe phenotype also presented migrating cells (Figure 6I, white arrowhead). The previous results suggest that ICAM ablation could increase the tumorigenic capabilities of the cells in situ.

### 2.5. ICAM1^KO^ Decreased the Metastatic Capabilities of GCSCs In Vivo

**Figure 7:**
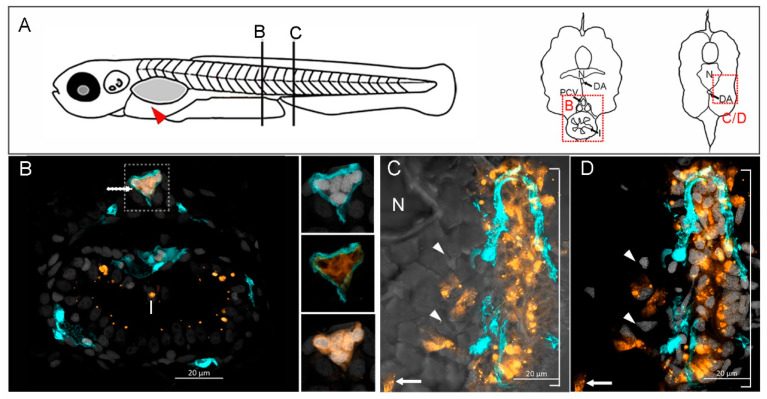
GCSCs migrate in groups and establish metastatic tumors. (**A**) Tg (fli1:EGFP)y1 zebrafish embryos (2 dpf) were given an injection into the yolk sac (red arrow) of 50 fluorescently observed migrating cells (white arrow) inside the posterior cardinal vein (PCV, cyan) over the distal portion of the intestine (I). The insets show five GCSC nuclei inside the PCV, co-stained with Hoechst (white). (**C**,**D**) The GCSCs formed a metastatic cell mass that invaded the skeletal muscle at the level of somites 21-21, as observed using a brightfield microscope (**C**), the nuclei were stained with Hoescht (**D**) to observe clearly the cell mass. The white arrows show GCSCs, and the white arrowheads indicate zebrafish muscle fiber nuclei. N indicates the notochord, DA the dorsal aorta, and I the intestine. The images were obtained at 63X with an LSM800 confocal microscope. Labeled GCSCs are shown in orange, and 6 µm sections are shown in the (**B**,**C**) insets. (**B**) GCSCs 6 dpi.

Of note, we were able to observe that GCSCs have the capability to migrate in groups through blood vessels. At 6 dpi, we observed a group of 9 to 10 cells (orange) in the posterior cardinal vein (PCV) (Figure 7A, inset B, and Figure 7B). It should be mentioned that the insets in Figure 7B show five cells; however, in the whole z-stack, we counted 9–10 GCSCs traveling inside this vessel. The GCSCs could migrate from the site of injection (yolk, red arrow in Figure 7A) to distal portions of the tail and invade the muscle at somites 20 to 21 (Figure 7A, inset C, and Figure 7B). In contrast, we did not observe GCSC/ICAM1^KO^ cells forming secondary tumors after migration; this indicates that ICAM ablation diminished migration and affected metastasis in vivo.

### 2.6. ICAM1 Knockout Increased the Resistance of GCSCs to Cisplatin In Vitro

**Figure 8:**
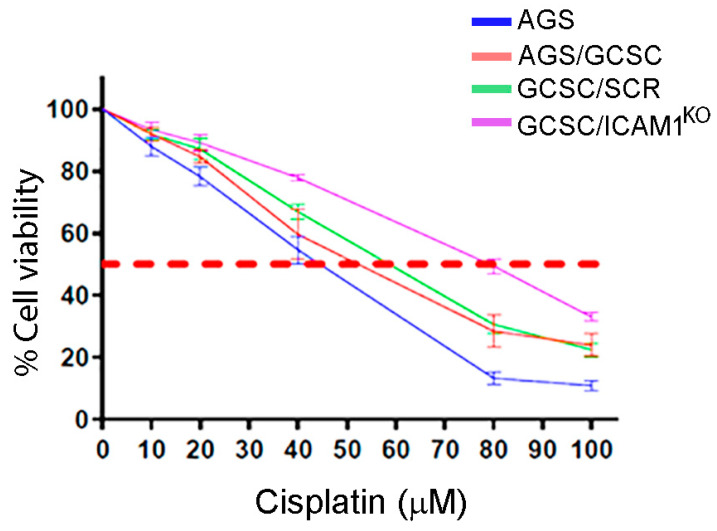
GCSC/ICAM1^KO^ cells exhibited an increased resistance to cisplatin. Viability percentages are shown for AGS (IC50 at 41.6 µM), AGS-GCSC (IC50 at 49.3 µM), GCSC-SCR (IC50 at 55.08 µM), and GCSC-ICAM1^KO^ (IC50 at 79.2 µM) cells in the presence of different concentrations of cisplatin. n = data from 3 independent assays. The dotted line means the 50% of cell viability.

To determine if ICAM1 modifies the chemoresistance of GCSCs to cisplatin, we evaluated the cell viability of GCSC/ICAM1^KO^ cells 24 h after treatment with cisplatin. The analyses showed that GCSC/ICAM1^KO^ cells have an increased chemoresistance to cisplatin, showing an IC50 of 49.3 µM (Table 1), compared to AGS, AGS/GCSC, and GCSC/SCR cells. This result suggests that ICAM1 could be related to the chemoresistance capacity of GCSCs (Figure 8).

## 3. Discussion

Previously, we described the presence of gastric cancer stem cells with the immunophenotype CD24+CD44+CD326+ICAM1+ in the gastric tissue of gastric cancer patients and some cell lines, including AGS. In vitro and in vivo assays demonstrated that these cells self-renew and have a high capability of migrating, invading, and metastasizing in a zebrafish model [14]. Interestingly, we found this subpopulation of circulating GCSCs in the blood samples of GC patients; this cell subpopulation was absent in healthy patients, in which we found the CD24+CD44+CD326+ cell subset, with the absence of the surface marker ICAM1. ICAM1 was expressed on the surface of cells with features such as chemoresistance, stemness marker expression, and tumor-initiating behavior [24,25,26]. In addition, the expression of ICAM1 is considered an inflammatory marker in cancer and is related to the migratory, invasive, and metastatic capacity of the many types of cancer cells [14,18,27]. In this work, we engineered stable GCSC/ICAM1^KO^ cells from the AGS cell line using CRISPR/iCas9 to analyze whether the expression of ICAM1 determines the stemness of these cells and, therefore, their ability to migrate, invade, and metastasize. In 2022, Lim et al. reported that ICAM1 forms a heterodimer with c-Met in colorectal cancer, triggering STAT3 activation in the c-Met/ICAM1/Src/STAT3 axis [23]. Once active, the transcription factor STAT3 enters the nucleus, where it can regulate the expression of some genes related to stemness [28] and generate positive feedback for ICAM1 [23], because the ICAM1 promoter has STAT3-binding domains. We show in Figure 4 that ICAM1 regulates STAT3 activation in GCSCs with the immunophenotype CD24+CD44+CD326+ICAM1+, according to a Western blot using the antibody anti-pSTAT3-Ser727. The activation of STAT3 by ICAM1 has been reported, but this activation also functions as a regulator of Src activation (SrcpY418) in endothelial cells during the leucocyte transendothelial migration [29]. The activity of the transcription factor STAT3 is involved in carcinogenesis and stemness due to its ability to regulate the expression of oncogenes, tumor suppressor genes, and stemness genes [30]. In this sense, the stemness has been attributed to the expression of stemness transcriptional factors, such as Nanog, Oct3/4, and Sox2. In this regard, the promoter of Nanog has a STAT3-binding domain that regulates its expression. Considering that the inter-regulation between stemness factors has long been proposed [31], the relationships among ICAM1/STAT3/Nanog support the idea that ICAM1 regulates CD24+CD44+CD326+ICAM1+ GCSC stemness. We showed that GCSC/ICAM1^KO^ cells exhibited a decreased expression of Nanog and Oct3/4 but not Sox2; we propose that the ICAM1/STAT3 axis is responsible for the downregulation of Nanog and Oct3/4 but with a possible function gain of Sox2 in these CD24+CD44+CD326+ICAM1+ GCSCs. However, it is necessary to delve deeper into this subject.

In gastrointestinal cancers, cells overexpressing stemness factors have been reported to have some characteristics similar to CSCs, such as their migration, invasion, tumorigenicity, and chemoresistance, which are present in the EMT. A relationship between CSCs and the EMT has been reported; in cancer, the EMT is associated with tumor initiation and resistance to chemotherapy but also invasion and metastasis [32]. For this reason, we analyzed the effects of ICAM1 on the ability of GCSCs to migrate and invade in vitro. Our results show that GCSC/ICAM1^KO^ cells lose their ability to migrate and invade in vitro. These results suggest changes in the expression of proteins related to the EMT, so we decided to analyze epithelial and mesenchymal markers such as E-cadherin, vimentin, and Zeb1. We showed that GCSC/ICAM1^KO^ cells do not lose their expression of epithelial and mesenchymal markers, supporting the hypothesis that CSCs may be in a hybrid state between the epithelial and mesenchymal states, as previously reported [33]. Our results demonstrate, through an in vivo analysis in zebrafish, that GCSCs can intravasate into the blood vessels of the zebrafish and migrate in clusters, supporting the hypothesis of a hybrid state in the EMT of CSCs. In this sense, the main function of ICAM1 is the extravasation of the lymphocytes at the inflammatory site in response to tissue damage; these actions are made possible by the interaction between ICAM1 and LFA1 [16]. This result indicates the importance of ICAM1 in cell migration, which is consistent with our previous observations.

Previously, we demonstrated that GCSCs xenotransplanted into zebrafish showed a high tumorigenic potential and a greater migration and invasion capacity for the formation of metastatic tumors in the distal portion of the intestine [14]. To analyze if GCSC/ICAM1^KO^ cells have the same tumor potential as GCSCs, we injected GCSC/ICAM1^KO^ cells into the yolk sac of zebrafish embryos at 2 dpf. We observed different types of phenotypes at 1 dpi—severe, moderate, and mild phenotypes (Figure 8)—with tumor formation in the yolk sac but with the disruption of the yolk sac in the embryos with a severe or moderate phenotype. This cell behavior was not observed in our previous reports. The cells were stained with CM-Dil, which allowed us to observe them using microscopy; we analyzed the presence of metastatic cells in a distal zone such as the one observed in our previous publication when we injected the GCSCs ga [14]. We also observed that the embryos injected with 50 GCSC/ICAM1^KO^ cells died earlier than the embryos injected with 50 GCSCs. These results reveal that ICAM1 in GCSCs confers a high capacity to migrate and invade distal regions to the xenotransplanted cells. Of note, GCSCs without ICAM1 exhibited decreased migration in xenotransplanted zebrafish, but they showed a more aggressive cell behavior, resulting in a tumoral mass in situ that was able to disrupt the structures in the yolk sac and cause a severe phenotype. These data suggest a great malignant potential for GCSC/ICAM1^KO^ cells after xenotransplantation in zebrafish embryos.

Our results demonstrate the crucial role of ICAM1 in regulating the metastatic capacity of GCSCs, potentially through STAT3 activation. This activation could be responsible for the downregulation of the Nanog and Oct3/4 stemness transcription factors. However, although the GCSC/ICAM1^KO^ cells exhibited a decreased expression of Nanog and Oct3/4, these cells did not lose their stemness features, possibly due to a gain of function through the stemness factor Sox2. These implications of our research could pave the way for novel therapeutic strategies targeting ICAM1 in GC.

Chemoresistance is one of the characteristics of CSCs. Interestingly, it has been reported that chemotherapy increases the CSC population in several types of cancer [8,34,35]. In this sense, prostate CSC chemoresistance increases ICAM1 expression after cisplatin treatment [28]. Therefore, ICAM1 is not only a CSC marker, but it also has a function in the acquisition of stemness. We also showed that GCSCs have more chemoresistance than the AGS cell line, but when we analyzed the chemoresistance of the GCSC-ICAM1^KO^ cells, we observed that these cells had more chemoresistance than the others. In contrast, it has been demonstrated that the prostate cancer PC3 cell knockdown of ICAM1 increased the sensitivity to cisplatin treatment, showing more apoptotic cells [28]. On the other hand, patients with gastric cancer who were treated with chemotherapy showed an enriched subpopulation of ICAM1+ cells that presented characteristics similar to cancer stem cells, as they can grow in spheroids in non-adherent conditions and because they demonstrated self-renewal and tumorigenic capabilities [8].

Our research findings not only shed light on the role of ICAM1 in regulating the metastatic capacity of GCSCs, but they also offer potential therapeutic strategies. We found that the downregulation of the Nanog and Oct3/4 stemness factors, possibly through STAT3 activation, was associated with the metastatic capacity of GCSCs. Interestingly, even though the GCSC/ICAM1^KO^ cells showed a decrease in the expression of these stemness transcription factors, they did not lose their stemness features, possibly due to a gain of function of the stemness factor Sox2. These findings suggest that targeting ICAM1 in GCSCs could be a promising therapeutic strategy, potentially inhibiting the metastatic capacity of GCSCs. However, upon further investigation, we observed that GCSC/ICAM1^KO^ GCSCs were more chemoresistant than GCSCs with the CD24+CD44+CD326+ICAM1+ immunophenotype. This highlights the complexity of gastric cancer and the need to carry out more studies that allow us to understand the role of cancer stem cells in the development and clinical outcomes of gastric cancer.

## 4. Materials and Methods

### 4.1. Materials and Chemical Reagents

The vectors Lenti-iCas9-neo (#85400), CROPseq-guide-puro (#86708), pCMV-dR8.2 dvpr (#8455), and pCMV-VSV-G (#8454) were purchased from Addgene (Watertown, MA, USA). The antibodies anti-CD24-PE (#338808), antiCD44-APC (#397506), anti-CD326-Pe-Cy7 (#234222), anti-ICAM1-Pacific blue (#353110), and permeabilization wash buffer (#421002) were purchased from BioLegend (San Diego, CA, USA). The serum knockout (#10820-028), fetal bovine serum (#26140079-PRO), B27 (#17504001), and neomycin (#10131-035) were purchased from Invitrogen^®^ (Waltham, MA, USA). Single-guide RNA (sgRNA) SCR and sgRNA anti-ICAM1 were purchased from IDT integrated DNA technologies^®^ (Coralville, IA, USA). The antibody anti-pSTAT3 (#9134) and STAT3 (#9139) were purchased from Cellsignal^®^ (Cell Signaling Technology, Inc., Danvers, MA, USA). The Lenti-X Concentrator was purchased from Clontech^®^ (#632165, Mountain View, CA, USA). Culture-insert 2 well dishes (Ibidi #80206, Fitchburg, WI, USA). Transwell 24-well plates containing a 6.5 mm insert with a 5.0 µm pore size polycarbonate membrane were purchased from Corning^®^ (#3421, Corning, NY, USA).

### 4.2. Cell Culture and Transduction

Monolayer cultures: The AGS gastric cancer and HEK293T cell lines were purchased from ATCC^®^ (Manassas, VA, USA). The AGS cells were maintained in F12 medium and HEK293T in DMEM. The F12 and DMEM media were supplemented with 10% fetal bovine serum, and the cells were then cultured at 37 °C with 5% CO_2_. The cells were sub-cultured with 0.02% EDTA.

Spheroid culture: AGS cells were maintained in serum-free F12 culture medium supplemented with 10% knockout serum, 1% B27, 10 ng/mL EGF (Invitrogen^®^, Waltham, MA, USA), 10 ng/ mL bFGF (Sigma Aldrich^®^, Darmstadt, Germany), penicillin (100 U/mL), and streptomycin (100 μg/mL). The AGS cells that expressed sgRNA-ICAM1-1 and sgRNA-ICAM1-2, as well as the AGS cells that expressed sgRNA-SCR, were grown in serum-free F12 medium supplemented with 500 µg/mL neomycin (Figure S1) and 1 µg/mL puromycin (Figure S2) in poly (2-hydroxyethyl methacrylate) (#P3932-25G, Sigma, Burlington, MA, USA)-treated dishes.

### 4.3. ICAM1 Knockout Using CRISPR/iCas9 System

The sgRNA (targeting ICAM1) and the sgRNA-SCR (scramble-sgRNA) unspecific sequence (Table S1) were obtained from the Origene portal and synthesized by IDTs (integrated DNA technologies). The sgRNAs were hybridized and cloned in the BSMBI site into the CROPseq-Guide-Puro vector and verified using SANGER sequencing (Figure S3), resulting in the plasmids pICAM1/1CROPseq, pICAM1/2CROPseq, and pSCR-CROPseq being obtained. To produce infectious lentiviral particles, we used the plasmids Lenti-iCas9-neo, pICAM1/1CROPseq, and pICAM1/2CROPseq or pSCR-CROPseq in independent experiments, together with the packing plasmids pCMV-dR8.2 dvpr and pCMV-VSV-G, which were transfected in a 5:5:1 ratio into HEK293T cells using the Lipofectamine^®^ 2000 Reagent (#1668027, Invitrogen^®^, Waltham, MA, USA) according to the manufacturer’s recommended protocol. The lentiviral supernatant was concentrated in a ratio of 10:1 using the Lenti-X Concentrator (Takara #621331, Kusatsu, Japan).

The transduction of the AGS cells was performed in a 6-well plate at a density of 100,000 cells/mL in a total volume of 2 mL of F12 medium supplemented with 10% FBS and 8 μg/mL polybrene (Sigma^®^ #H9268-5G). The volume of the lentiviral supernatant (Lenti-iCas9-neo) required for each experiment was added. The AGS/GFPiCas9 cells were grown in 10% FBS in F12 medium supplemented with 500 μg/mL neomycin. For a transduction efficiency assessment, AGS/iCas9GFP cells were treated with 1 μg/mL doxycycline for 24 h, and the percentage of GFP-positive cells was assessed using flow cytometry. Cell sorting was performed on cells with GFP reporter expression, which were harvested for subsequent seeding in F12 medium supplemented with 10% FBS. The data were analyzed using the Flow Jo 10.0 software^®^.

Spheroids were grown under non-adherent conditions with AGS/iCas9GFP cells in a serum-free culture medium supplemented with neomycin at 500 μg/mL to enrich the stem population. After three days of culture, gastric cancer stem cells were sorted to obtain those with the CD24+CD44+CD326+ICAM1+ immunophenotype; these cells were transduced with the pICAM1/1CROPseq and pICAM1/2CROPseq or pSCR-CROPseq plasmids, following the conditions previously described. Cell sorting was performed to isolate the CD24+CD44+CD326+ICAM1− cells. To assess transduction efficiency, the cells were treated with doxycycline at a concentration of 1 μg/mL for 48 h. The percentage of GFPiCas9+CD24+CD44+CD326+ICAM1− cells was determined using flow cytometry. The GCSC/ICAM1^KO^ and the GCSC/SCR cells were harvested and maintained in serum knockout F12 medium supplemented with 500 μg/mL neomycin and 1 μg/mL puromycin.

### 4.4. Flow Cytometry

For the cell surface staining, we performed a multi-parametric staining using 2 µL per million cells of the following antibodies: CD24, CD44, CD326, and ICAM1. After 20 min of incubation at room temperature, the cells were rinsed twice with 0.5% BSA in PBS. According to the manufacturer’s instructions, the cells were permeabilized with a permeabilization wash buffer for intracellular staining. The cells were acquired on a FACSAria II cytometer at the “Laboratorio Nacional CONAHCYT de Investigación y Diagnóstico por Inmunocitofluorometría” (LANCIDI), INER, México. We acquired at least 1 × 10^5^ events for the experiment. The acquisition data were analyzed using the Flow Jo software. For the cell-sorting assays, the cells were stained and sorted with flow cytometry using a FACSAria II cytometer. A post-sort analysis was performed each time to ensure that the purity of the cell fractions was >95%. The cells were recovered in the serum knockout, washed twice with sterile PBS, and counted before reseeding or injecting them into zebrafish embryos.

### 4.5. Wound-Healing Migration Assay

AGS, GCS/ICAM1^KO^, and GCSC/SCR cells were grown on a 2-well culture-insert in a 35 mm µ-Dish until the insert reached 100% confluence. The cells were treated for 2 h with 10 μg/mL of mitomycin C to inhibit proliferation. After the treatment, the cell monolayer was scratched and wounded, the insert was removed and washed twice with 1X PBS to remove the detached cells, and the cells were refreshed with serum-free F12 medium. The cells were incubated for 48 h at 37 °C in a 5% CO_2_ atmosphere. Following incubation, phase-contrast images were acquired at 24 and 48 h using an OLYMPUS IX51 microscope (Evident, Mexico City, Mexico) with a 4x objective and analyzed with the ImageJ software (version 1.54).

### 4.6. Transwell Invasion Assay

Matrigel invasion assays were performed by following the transwell chamber method, in 24-well plates containing a 6.5 mm insert with a 5.0 µm pore size polycarbonate membrane. The Matrigel (Corning^®^ 356234) was added in a 1:10 ratio of Matrigel to F12 medium to the top side of the inserts and incubated at 37 °C for 2 h to acquire a semisolid matrix. A total of 1 × 10^5^ cells per insert were seeded on the top side of the insert in 150 µL of serum-free F12 medium. The lower chamber was filled with 600 µL of F12 supplemented with 10% FBS. The cells were incubated for 48 h. After 48 h, the cells and Matrigel on the upper surface of the transwell insert were gently removed with cotton swabs. The invading cells on the lower surface of the membrane were washed with 1X PBS, fixed with 4% paraformaldehyde, and stained with 0.1% crystal violet diluted in 1X PBS. Images of the invading cells were acquired using an OLYMPUS IX51 microscope with a 20× objective and analyzed with the ImageJ software.

### 4.7. Western Blot

The total protein extract from the monolayer and spheroids cells was obtained by lysing the cells with RIPA buffer (50 mM tris-HCl, a pH of 7.4, 150 mM NaCl, 1 mM EDTA, 0.5% sodium deoxycholate, 1% NP-40, 0.1% SDS, 10 mM NaF, 1 mM PMSF, and 1 mM Na_3_VO_4_). The proteins were separated using 10% SDS–polyacrylamide gel electrophoresis (SDS-PAGE) and transferred to nitrocellulose membranes (Bio-Rad, Hercules, CA, USA). The membranes were blocked with 50 mg/mL of nonfat dry milk for 1 h and incubated overnight at 4 °C with the appropriate primary antibodies. The membranes were incubated with an HRP-conjugated secondary antibody for 2 h at room temperature. Detection was achieved using the SuperSignal Kit (Pierce, Rockford, IL, USA) in a C-DiGit Blot scanner (LI-COR Biosciences, Lincoln, NE, USA), and the results were analyzed using the Image Studio™ Lite v5.2 software (LI-COR Biosciences).

### 4.8. Zebrafish Husbandry and Lines

Adult zebrafish (*Danio rerio*) were maintained at 28.5 °C in the aquarium facility of Dr. Fernando López-Casillas at the Instituto de Fisiología Celular, UNAM (IFC, UNAM), according to standard procedures [36]. Dr. Jesus Torres Vazquez kindly donated the wild-type and transgenic zebrafish lines from the Department of Cell Biology, NYU Grossman School of Medicine, USA. Zebrafish embryos were obtained from natural crosses; we placed 1 male and 1 female adult zebrafish (6 to 18 months old) in an individual rearing tank. All the experiments were approved by the Committee for Laboratory Animal Care and Use of the IFC, UNAM, under CICUAL-protocol number FLC139-18.

Transgenic zebrafish embryos, Tg (fli1:EGFP)y1, expressing EGFP in their endothelial cells were staged based on the number of hours post-fertilization (hpf) or days post-fertilization (dpf) according to Kimmel et al., 1995 [37]. The zebrafish embryos were treated with phenylthiourea (PTU; 0.003% *w*/*v*; Sigma) to prevent the pigmentation of the larvae. All the animals were anesthetized with 164 mg/L tricaine (MS-222, Sigma) before euthanasia, which was performed by chilling on ice.

### 4.9. Microinjection of GCSCs into Zebrafish Embryos

Tumorsphere cells derived from the AGS-GCSC, GCSC-ICAM1^KO^, and GCSC-SCR cell lines were sorted in a fluorescence flow cytometer (FACSAria II). Then, the cells were stained with 1 µg/mL of CM-DiI dye (Invitrogen, Life Technologies) according to the manufacturer’s instructions and resuspended in fresh 1X PBS. The suspension’s cell density was measured with a hemocytometer and adjusted to 50 × 10^6^ cells/mL. The cell viability was verified using trypan blue staining.

On the day of the injection, 48 hpf Tg (fli1:EGFP)y1 zebrafish embryos were dechorionated and randomly separated into 4 groups (n = 60 to 80), described as follows: (1) AGS/GCSC, CD24+CD44+CD326+ICAM1+; (2) GCSC/ICAM1^KO^, CD24+CD44+CD326+ICAM1−; (3) GCSC/SCR, CD24+CD44+CD326+ICAM1+; and (4) uninjected control embryos. The embryos were anesthetized, and cells were injected into their yolk sac for all the experiments according to the order described above. After the cell injection (xenotransplantation), the four groups of embryos were placed in 100 mm Petri dishes with fresh water and incubated at 31 °C. Two to three hours post-injection (hpi), the embryos with cells in circulation or mechanical damage were discarded. Photographs or measurements were recorded from 15:00 to 19:00 h every day, starting on the day after the injection.

For each experiment, 50 cells were injected into the yolk sac using a microinjector (Femtojet express, Eppendorf, Hamburg, Germany) and a stereoscopic microscope (SMZ 745T, Nikon, Tokyo, Japan). After the injection, the embryos were incubated at 31 °C to allow for the growth of the injected cells and the zebrafish embryos [38].

### 4.10. Histological Processing

Anesthetized larvae were fixed with 4% paraformaldehyde in PBS-T overnight at 4 °C, washed 3 times in PBS-T for 10 min, and embedded in 15% sucrose–7.5% gelatin in PBS for cryosectioning (Leica^®^, Buffalo Grove, IL, USA). Transverse sections of 6, 10, or 15 µm were obtained and mounted for the direct observation of fluorescent cells or processed for either hematoxylin and eosin (H&E) staining or periodic acid–Schiff/Alcian blue staining (PAS-AB). The staining was performed in the histology facilities of the IFC and UNAM. The tissue sections for fluorescence image acquisition were stained with Hoechst dye to observe the nuclei.

### 4.11. Imaging

We monitored the in vivo tumor formation and cell migration of the injected fluorescently labeled cells from 1 to 6 dpi on a Nikon SMZ1500 stereomicroscope. The images of whole zebrafish larvae were acquired daily with a DS-Fi1 camera (Nikon) and NIS Elements F software v4.3 (Nikon). First, the image background was subtracted from each channel, and then the overlay was performed with the FIJI (ImageJ) software. For whole-embryo images, we increased the signal intensity of the stained GCSCs to make them visible at 2X magnification. Fluorescence images of whole larvae or cryosections were acquired with an LSM 800 confocal microscope (batch number 2633000222, Carl Zeiss, Oberkochen, Germany) with GaAsP detectors and a Plan-Apochromat 20X/0.8 M27, Plan-Apochromat 40X/1.3 oil DIC (UV) VIS-IR M27, or Plan-Apochromat 63X/1.4 oil DIC M27 objective. Image acquisition and processing were performed using the Carl Zeiss Zen Blue 2.3 software. We acquired tiled array images of 2980 × 4914 pixels per image with a 20X objective, and then we extracted single-slice images for the figures presented in this report. No processing was applied to the images included in this report, and we only enhanced the signal at the same level for each channel to visualize the images easily. Images acquired with a 63X objective were acquired as single images or a tiled array of images with 1437 × 1437 pixels per image. No processing was applied, and we only enhanced the signal at the same level for each channel.

Image acquisition for the H&E- or AB-Pas-stained slides was carried out on a stereoscopic microscope AxioZoom V16 with an ApoTome (Carl Zeiss, batch number: 4633001353) and a PlanNeoFluar Z 2.3X/0.57 objective. Image acquisition was performed with the Axiocam503 and Zen PRO software (Carl Zeiss). The figures were exported to Photoshop Cs6 (Adobe) for final editing and presentation.

### 4.12. Statistical Analysis

All the statistical results are expressed as the mean and standard error of the mean (SEM) using GraphPad Prism 5.0. Decreases/increases in fold changes were analyzed using a one-way ANOVA. All the experiments were repeated at least three times.

## 5. Conclusions

The search for therapeutic targets against CSCs has intensified due to the role of CSCs in promoting tumor growth and resistance to chemotherapy. Our research has identified ICAM1 as a possible marker of CSCs. We previously demonstrated the distinct expression of ICAM1 between gastric cancer patients and healthy volunteers, suggesting its potential as a therapeutic target. However, our current work reveals a challenging aspect: the disruption of ICAM1 decreases the ability of GCSCs to migrate, invade, and metastasize. However, it also amplifies their chemoresistance and tumorigenicity, leading to a more malignant cell population. These findings highlight the intricate nature of CSC therapy and the potential consequences of failed treatments, underscoring the urgency to carry out research that allows us to better address this complex problem.

## Figures and Tables

**Table 1:** IC50 calculated for the different cell lines.

Cell Line	Cisplatin IC50 µM
AGS	41.6 ± 2.9
AGS-GCSC	49.3 ± 6.9
GCSC-SCR	55.08 ± 2.8
GCSC-ICAM1^KO^	79.2 ± 2.9

## Data Availability

The original contributions presented in this study are included in this article/Supplementary Material; further inquiries can be directed to the corresponding author.

## Supplementary Materials

The following supporting information can be downloaded at https://www.mdpi.com/article/10.3390/ijms25168865/s1.

## References

[1] Thrift A.P., El-Serag H.B. (2020). Burden of Gastric Cancer. Clin. Gastroenterol. Hepatol..

[2] Smyth E.C., Nilsson M., Grabsch H.I., Van Grieken N.C., Lordick F. (2020). Gastric Cancer. Lancet.

[3] Sung H., Ferlay J., Siegel R.L., Laversanne M., Soerjomataram I., Jemal A., Bray F. (2021). Global Cancer Statistics 2020: GLOBOCAN Estimates of Incidence and Mortality Worldwide for 36 Cancers in 185 Countries. CA Cancer J. Clin..

[4] Yang L., Shi P., Zhao G., Xu J., Peng W., Zhang J., Zhang G., Wang X., Dong Z., Chen F. (2020). Targeting Cancer Stem Cell Pathways for Cancer Therapy. Signal Transduct. Target. Ther..

[5] Singh S.K., Clarke I.D., Terasaki M., Bonn V.E., Hawkins C., Squire J., Dirks P.B. (2003). Identification of a Cancer Stem Cell in Human Brain Tumors. Cancer Res..

[6] Al-Hajj M., Wicha M.S., Benito-Hernandez A., Morrison S.J., Clarke M.F. (2003). Prospective Identification of Tumorigenic Breast Cancer Cells. Proc. Natl. Acad. Sci. USA.

[7] O’Brien C.A., Pollett A., Gallinger S., Dick J.E. (2007). A Human Colon Cancer Cell Capable of Initiating Tumour Growth in Immunodeficient Mice. Nature.

[8] Bagheri V., Memar B., Behzadi R., Aliakbarian M., Jangjoo A., Bahar M.M., Talebi S., Gholamin M., Abbaszadegan M.R. (2018). Isolation and Identification of Chemotherapy-enriched Sphere-forming Cells from a Patient with Gastric Cancer. J. Cell. Physiol..

[9] Zhou T., Liu J., Xie Y., Yuan S., Guo Y., Bai W., Zhao K., Jiang W., Wang H., Wang H. (2022). ESE3/EHF, a Promising Target of Rosiglitazone, Suppresses Pancreatic Cancer Stemness by Downregulating CXCR4. Gut.

[10] Zheng H., Liu H., Li H., Dou W., Wang J., Zhang J., Liu T., Wu Y., Liu Y., Wang X. (2022). Characterization of Stem Cell Landscape and Identification of Stemness-Relevant Prognostic Gene Signature to Aid Immunotherapy in Colorectal Cancer. Stem Cell Res. Ther..

[11] Xiao Y., Chen J., Wang J., Guan W., Wang M., Zhang L., Wang Z., Wang L., Yu L. (2021). Acute Myeloid Leukemia Epigenetic Immune Escape From Nature Killer Cells by ICAM-1. Front. Oncol..

[12] Alvarado-Ortiz E., Sarabia-Sánchez M.Á., García-Carrancá A. (2019). Molecular Mechanisms Underlying the Functions of Cellular Markers Associated with the Phenotype of Cancer Stem Cells. Curr. Stem Cell Res. Ther..

[13] Walcher L., Kistenmacher A.-K., Suo H., Kitte R., Dluczek S., Strauß A., Blaudszun A.-R., Yevsa T., Fricke S., Kossatz-Boehlert U. (2020). Cancer Stem Cells—Origins and Biomarkers: Perspectives for Targeted Personalized Therapies. Front. Immunol..

[14] Gómez-Gallegos A.A., Ramírez-Vidal L., Becerril-Rico J., Pérez-Islas E., Hernandez-Peralta Z.J., Toledo-Guzmán M.E., García-Carrancá A., Langley E., Hernández-Guerrero A., López-Casillas F. (2023). CD24+CD44+CD54+EpCAM+ Gastric Cancer Stem Cells Predict Tumor Progression and Metastasis: Clinical and Experimental Evidence. Stem Cell Res. Ther..

[15] Luo B.-H., Carman C.V., Springer T.A. (2007). Structural Basis of Integrin Regulation and Signaling. Annu. Rev. Immunol..

[16] Bui T.M., Wiesolek H.L., Sumagin R. (2020). ICAM-1: A Master Regulator of Cellular Responses in Inflammation, Injury Resolution, and Tumorigenesis. J. Leukoc. Biol..

[17] Ramos T.N., Bullard D.C., Barnum S.R. (2014). ICAM-1: Isoforms and Phenotypes. J. Immunol..

[18] Qiu Z., Wang Y., Zhang Z., Qin R., Peng Y., Tang W., Xi Y., Tian G., Zhang Y. (2022). Roles of Intercellular Cell Adhesion Molecule-1 (ICAM-1) in Colorectal Cancer: Expression, Functions, Prognosis, Tumorigenesis, Polymorphisms and Therapeutic Implications. Front. Oncol..

[19] Kuppner M.C., Meir E.V., Hamou M.F., Tribolet N.D. (2008). Cytokine Regulation of Intercellular Adhesion Molecule-1 (ICAM-1) Expression on Human Glioblastoma Cells. Clin. Exp. Immunol..

[20] Sawa Y., Ueki T., Hata M., Iwasawa K., Tsuruga E., Kojima H., Ishikawa H., Yoshida S. (2008). LPS-Induced IL-6, IL-8, VCAM-1, and ICAM-1 Expression in Human Lymphatic Endothelium. J. Histochem. Cytochem..

[21] Ohh M., Smith C.A., Carpenito C., Takei F. (1994). Regulation of Intercellular Adhesion Molecule-1 Gene Expression Involves Multiple mRNA Stabilization Mechanisms: Effects of Interferon-Gamma and Phorbol Myristate Acetate. Blood.

[22] Schardt C., Heymanns J., Schardt C., Rotsch M., Havemann K. (1993). Differential Expression of the Intercellular Adhesion Molecule-1 (ICAM-1) in Lung Cancer Cell Lines of Various Histological Types. Eur. J. Cancer.

[23] Lim E.-J., Kang J.-H., Kim Y.-J., Kim S., Lee S.-J. (2022). ICAM-1 Promotes Cancer Progression by Regulating SRC Activity as an Adapter Protein in Colorectal Cancer. Cell Death Dis..

[24] Taftaf R., Liu X., Singh S., Jia Y., Dashzeveg N.K., Hoffmann A.D., El-Shennawy L., Ramos E.K., Adorno-Cruz V., Schuster E.J. (2021). ICAM1 Initiates CTC Cluster Formation and Trans-Endothelial Migration in Lung Metastasis of Breast Cancer. Nat. Commun..

[25] Figenschau S.L., Knutsen E., Urbarova I., Fenton C., Elston B., Perander M., Mortensen E.S., Fenton K.A. (2018). ICAM1 Expression Is Induced by Proinflammatory Cytokines and Associated with TLS Formation in Aggressive Breast Cancer Subtypes. Sci. Rep..

[26] Tsai S.-T., Wang P.-J., Liou N.-J., Lin P.-S., Chen C.-H., Chang W.-C. (2015). ICAM1 Is a Potential Cancer Stem Cell Marker of Esophageal Squamous Cell Carcinoma. PLoS ONE.

[27] Zhang L., Dong B., Yuan X. (2022). Expression of ALDH1 Plays the Important Role during Generation and Progression in Human Cervical Cancer. Biotechnol. Genet. Eng. Rev..

[28] Li T., Wang Z., Hou Y., Li Y. (2017). Pim-3 Regulates Stemness of Pancreatic Cancer Cells via Activating STAT3 Signaling Pathway. J. Cancer.

[29] Allingham M.J., Van Buul J.D., Burridge K. (2007). ICAM-1-Mediated, Src- and Pyk2-Dependent Vascular Endothelial Cadherin Tyrosine Phosphorylation Is Required for Leukocyte Transendothelial Migration. J. Immunol..

[30] Li C., Liu S., Yan R., Han N., Wong K.-K., Li L. (2017). CD54-NOTCH1 Axis Controls Tumor Initiation and Cancer Stem Cell Functions in Human Prostate Cancer. Theranostics.

[31] Li M., Izpisua Belmonte J.C. (2018). Deconstructing the Pluripotency Gene Regulatory Network. Nat. Cell Biol..

[32] Puisieux A., Brabletz T., Caramel J. (2014). Oncogenic Roles of EMT-Inducing Transcription Factors. Nat. Cell Biol..

[33] Pastushenko I., Brisebarre A., Sifrim A., Fioramonti M., Revenco T., Boumahdi S., Van Keymeulen A., Brown D., Moers V., Lemaire S. (2018). Identification of the Tumour Transition States Occurring during EMT. Nature.

[34] Ma S., Lee T.K., Zheng B.-J., Chan K.W., Guan X.-Y. (2008). CD133+ HCC Cancer Stem Cells Confer Chemoresistance by Preferential Expression of the Akt/PKB Survival Pathway. Oncogene.

[35] Lee T.K.W., Castilho A., Cheung V.C.H., Tang K.H., Ma S., Ng I.O.L. (2011). CD24+ Liver Tumor-Initiating Cells Drive Self-Renewal and Tumor Initiation through STAT3-Mediated NANOG Regulation. Cell Stem Cell.

[36] Westerfield M. (2000). The Zebrafish Book: A Guide for the Laboratory Use of Zebrafish (Danio Rerio).

[37] Kimmel C.B., Ballard W.W., Kimmel S.R., Ullmann B., Schilling T.F. (1995). Stages of Embryonic Development of the Zebrafish. Dev. Dyn..

[38] Wertman J., Veinotte C.J., Dellaire G., Berman J.N., Langenau D.M. (2016). The Zebrafish Xenograft Platform: Evolution of a Novel Cancer Model and Preclinical Screening Tool. Cancer and Zebrafish.

