# Supplementary material for: ICAM1 (CD54) Contributes to the Metastatic Capacity of Gastric Cancer Stem Cells
Source: Int J Mol Sci. 2024 Aug 14;25(16):8865. doi: 10.3390/ijms25168865 (PMC11354656; doi:10.3390/ijms25168865)
Supplement: Supplementary file 1 [file ijms-25-08865-s001.zip › ijms-3125237-supplementary.pptx]

## Slide 1
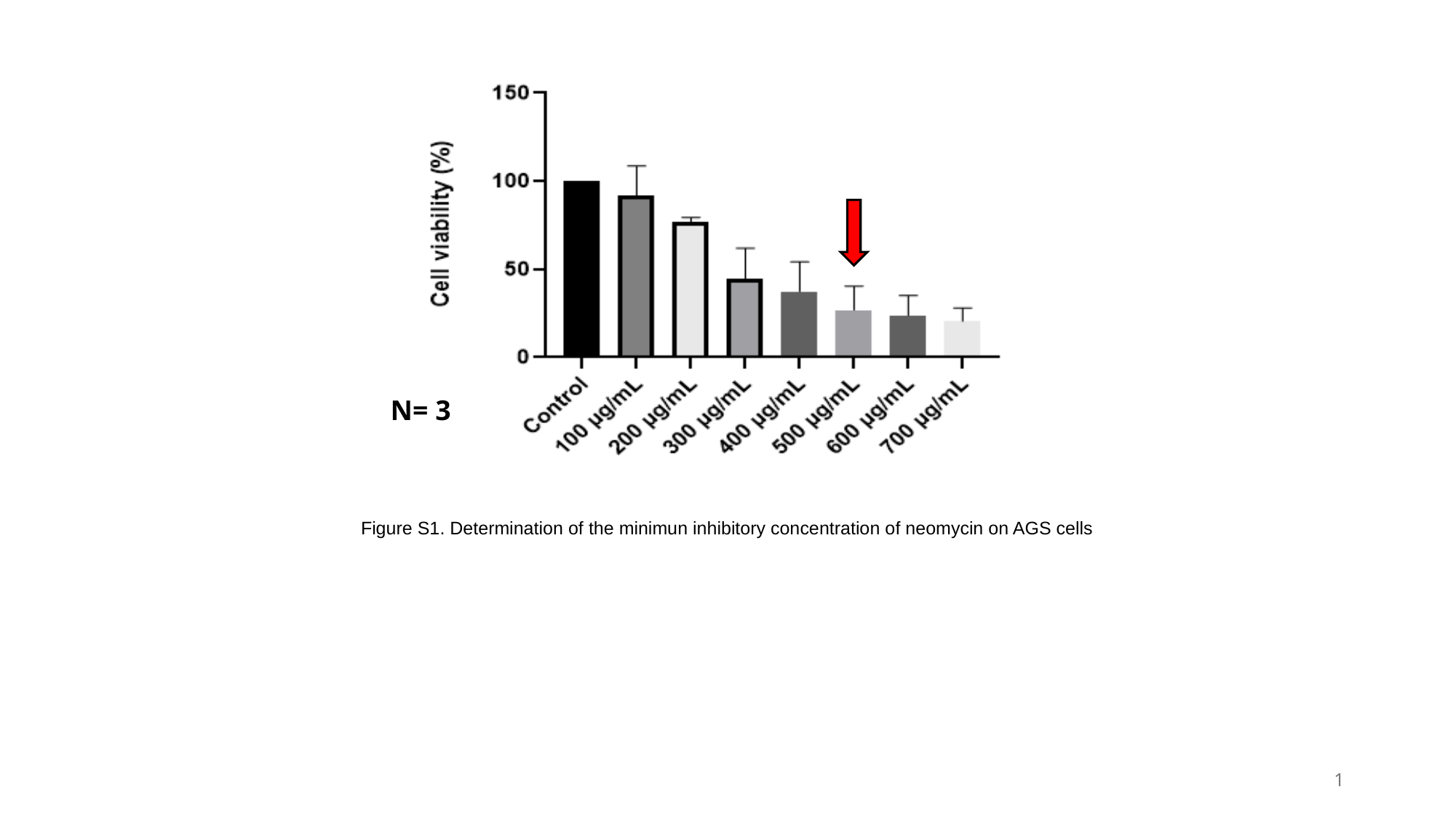

N= 3
# Figure S1. Determination of the minimun inhibitory concentration of neomycin on AGS cells
1

## Slide 2
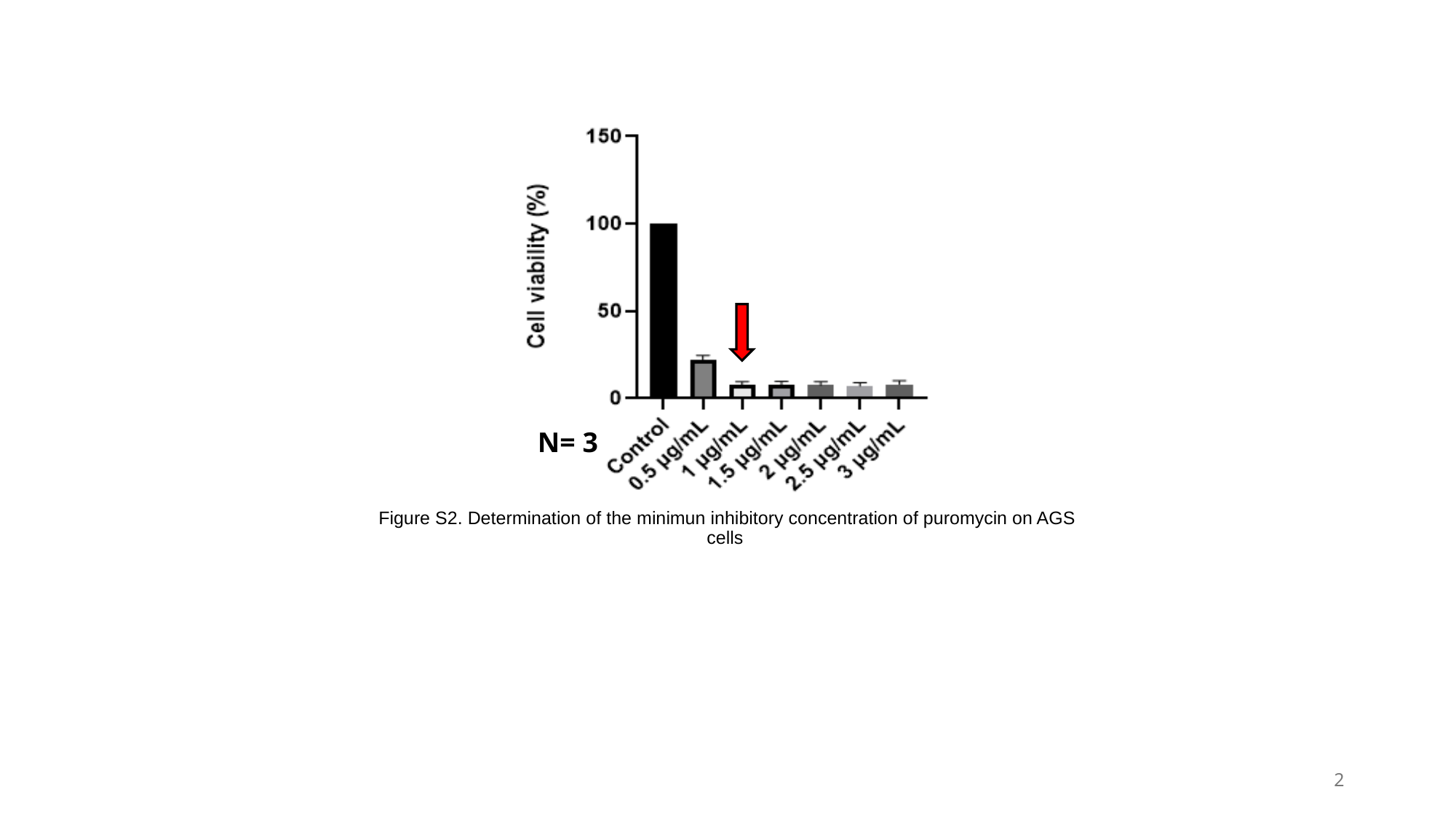

N= 3
# Figure S2. Determination of the minimun inhibitory concentration of puromycin on AGS cells
2

## Slide 3
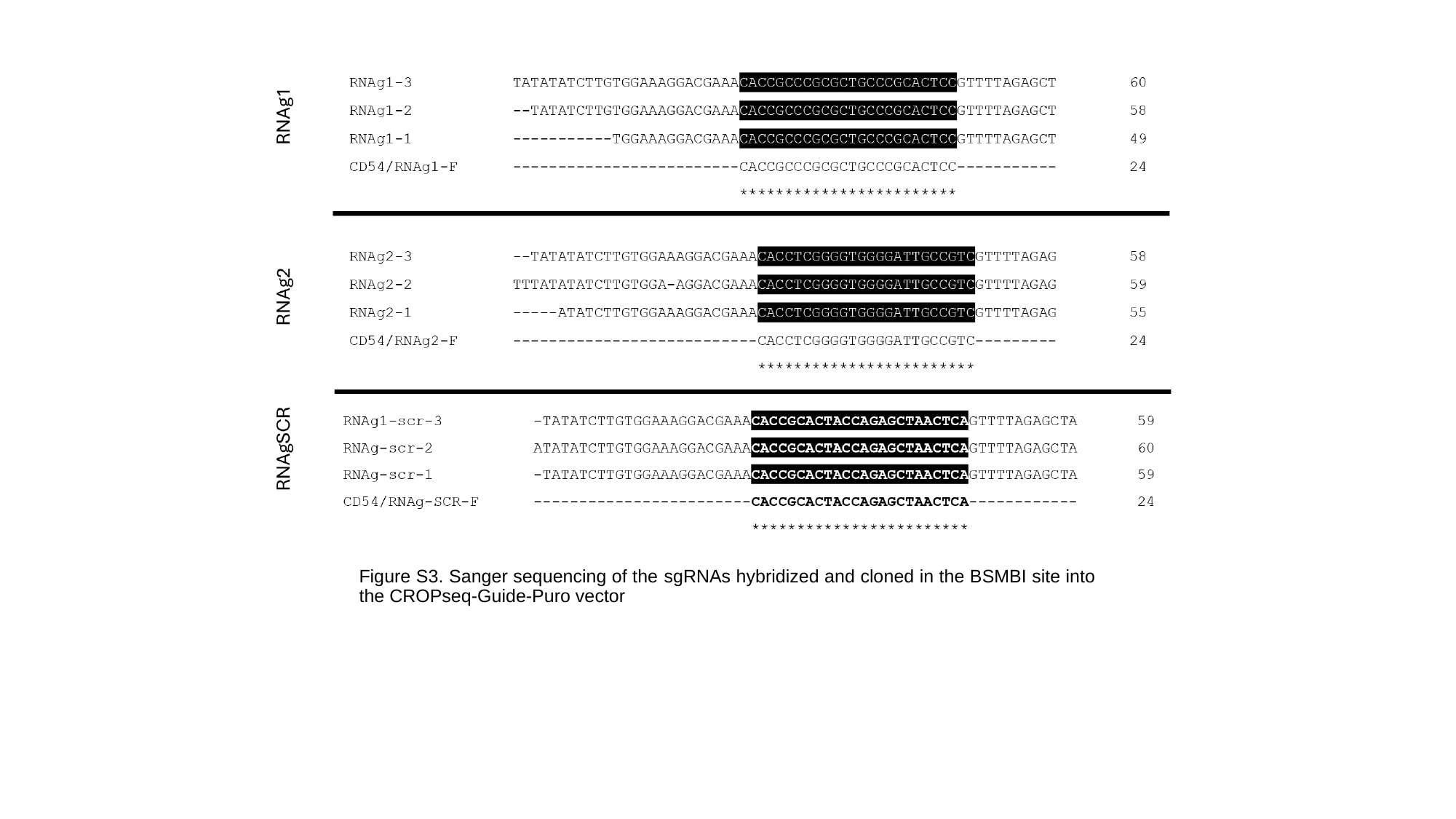

# Figure S3. Sanger sequencing of the sgRNAs hybridized and cloned in the BSMBI site into the CROPseq-Guide-Puro vector

## Slide 4
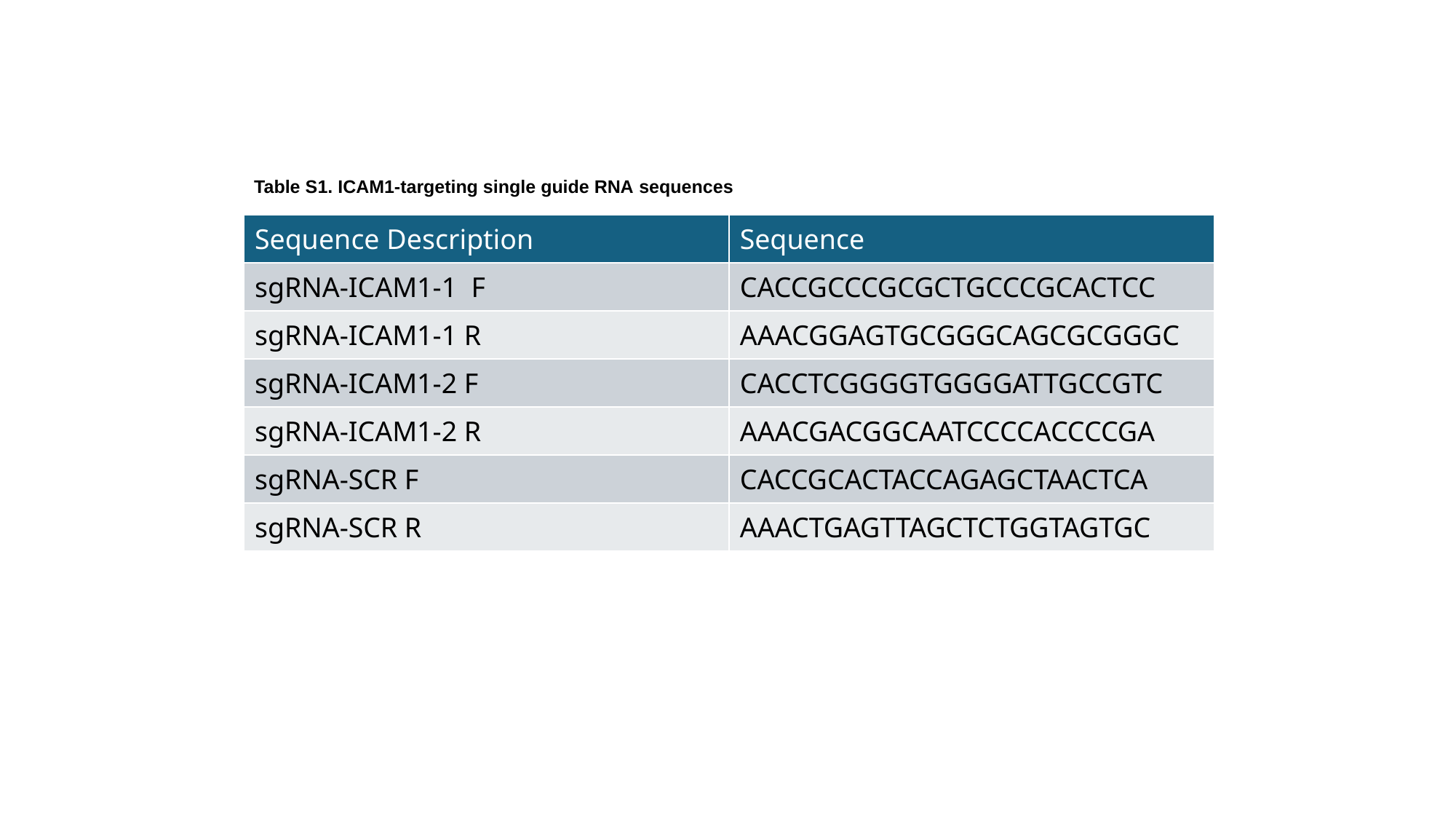

Table S1. ICAM1-targeting single guide RNA sequences
| Sequence Description | Sequence |
| --- | --- |
| sgRNA-ICAM1-1 F | CACCGCCCGCGCTGCCCGCACTCC |
| sgRNA-ICAM1-1 R | AAACGGAGTGCGGGCAGCGCGGGC |
| sgRNA-ICAM1-2 F | CACCTCGGGGTGGGGATTGCCGTC |
| sgRNA-ICAM1-2 R | AAACGACGGCAATCCCCACCCCGA |
| sgRNA-SCR F | CACCGCACTACCAGAGCTAACTCA |
| sgRNA-SCR R | AAACTGAGTTAGCTCTGGTAGTGC |
